# Association of Virtual Away Rotations With Residency Applicant Outcomes in Otolaryngology

**DOI:** 10.1002/oto2.78

**Published:** 2023-09-08

**Authors:** Nicholas R. Lenze, William J. Benjamin, Lauren A. Bohm, Marc C. Thorne, Michael J. Brenner, Angela P. Mihalic, Robbi A. Kupfer

**Affiliations:** ^1^ Department of Otolaryngology–Head and Neck Surgery University of Michigan Medical School Ann Arbor Michigan USA; ^2^ Department of Pediatrics University of Texas Southwestern Medical Center Dallas Texas USA

**Keywords:** graduate medical education, otolaryngology, residency application, virtual rotations, pandemic

## Abstract

**Objective:**

To examine how virtual away rotations might influence interview and match outcomes in otolaryngology.

**Study Design:**

Cross‐sectional retrospective analysis of survey‐based study.

**Setting:**

United States medical students applying to otolaryngology residency in the 2020 to 2021 cycle.

**Methods:**

The Texas Seeking Transparency in Application to Residency database was queried to identify otolaryngology applicants during the 2020 to 2021 cycle. The primary outcome was mean number of interview offers. *χ* ^2^ tests, 2‐sided *t* tests, logistic regression models, and ordinary least squares regression models were used to examine associations with virtual away rotations.

**Results:**

Among 115 otolaryngology applicants identified, 35 (30.4%) applicants reported completing 1 or more virtual away rotations. Applicants who completed at least 1 virtual away rotation received significantly more interview offers than their counterparts who did not participate in virtual away rotations (mean [SD], 14.9 [8.2] vs 11.6 [7.9]; *P* < .03). Each virtual away rotation completed was associated with an incremental increase of 2 additional interview offers (*β* coefficient: 2.29 [95% confidence interval, CI: 0.8‐3.7; *P* < .01]). Applicants who completed a virtual away rotation were more likely to receive an interview from that program (62.7% vs 16.8%, *P* < .01) and to match there (odds ratio 7.7 [95% CI: 2.7‐21.7]; *P* < .01) when compared to applicants who had not done the away rotation. Participation in virtual away rotations was not associated with significant improvement in match success (82.9% vs 67.5%; *P* = .09).

**Conclusion:**

Virtual away rotations were associated with improved program‐specific interview and match outcomes, as well as a higher overall number of interview offers.

Restrictions on in‐person activities during the early COVID‐19 pandemic spurred innovations in medical education that may have ongoing relevance to the residency application process. Virtual away rotations were designed by some programs as an alternative to the traditional in‐person away rotations, which have historically been important for matching into otolaryngology.^1^, ^2^ Prior to the COVID‐19 pandemic, away rotations allowed otolaryngology applicants to showcase their skills and foster valuable connections with programs, likely increasing their chances of receiving interviews at programs of interest.

A longstanding concern regarding in‐person away rotations is the significant cost burden borne by medical student applicants and the potential for creating inequities in the interview process. These costs might disproportionately dissuade applicants from backgrounds underrepresented in medicine (URiM).^3^, ^4^ Applicant‐reported data from the 2018 to 2020 match years in otolaryngology suggest an average spending of about $2437 for away rotations per applicant over the course of an application cycle.^5^ Addressing the cost burden of away rotations could improve the pathway for URiM applicants in otolaryngology, which has significant race‐ and sex‐based disparities in resident selection and faculty promotion.^6^, ^7^, ^8^, ^9^

Although virtual away rotations cannot replace the experience gained from in‐person clinical rotations, they can allow applicants to build their knowledge base, showcase their skills, and evaluate their fit with a residency program without incurring the expenses of in‐person away rotations. Given the potential role of virtual away rotations in future residency recruitment and selection cycles, further investigation into their effectiveness is warranted. We, therefore, investigated whether virtual away rotations were associated with interview and match outcomes in the 2021 otolaryngology match using the Texas Seeking Transparency in Residency (Texas STAR) database. We hypothesized that virtual away rotations would be associated with an increased likelihood of interviewing and matching at the site of an away rotation.

## Methods

This database study was reviewed by the Institutional Review Board (IRB) at the University of Michigan and deemed exempt on the grounds that it did not constitute human subjects research (IRB #HUM00217169).

### Sample Selection

Otolaryngology applicants who participated in the 2021 otolaryngology residency match and responded to the Texas STAR survey were included. The survey was distributed by the student affairs dean at participating medical schools, and it was available for students to complete between match day and April 5, 2021. The Texas STAR survey was distributed to participating allopathic and osteopathic medical schools in the United States. Data on international medical graduate (IMG) outcomes were not available for this analysis because the Texas STAR survey was not distributed to international medical schools.

### Texas STAR Survey and Variables

The survey asked applicants to report information as it would have appeared on their residency application. Demographic information such as age, sex, and race was not collected. US Medical Licensing Examination (USMLE) step 1 and step 2 Clinical Knowledge scores were reported within a 5‐digit range (ie, 220‐224) to help protect applicant confidentiality. The survey presented respondents with a list of programs from the Electronic Residency Application Service (ERAS), and for each program, respondents could designate whether they completed a virtual away rotation, received an interview offer, or matched at that program.

### Statistical Analysis

The primary outcome was the mean number of interview offers. Secondary outcomes included interview rate (Total interview offers/Total applications submitted × 100) and match success. We assessed differences in applicant characteristics, interview offers, and match outcomes between those who did and did not complete a virtual away rotation using *χ* ^2^ tests and Wilcoxon‐rank sum tests. We used ordinary least square (OLS) regression modeling to assess the association of virtual away rotations with the total number of interview offers. We used univariate logistic regression models to assess the relationship between the number of virtual away rotations and the odds of match success. We performed an application‐level analysis to examine the impact of receiving an interview offer or matching at a specific program where a virtual away rotation was completed. We set a significance criterion of *P* < .05 for all testing, and we used SAS version 9.4 for all analyses.

## Results

### Applicant Characteristics

There were 115 otolaryngology applicants who participated in the 2021 residency match and completed the Texas STAR survey. Among these, 35 (30.4%) applicants completed at least 1 virtual away rotation. Among the 35 applicants who completed at least 1 virtual away rotation, 14 (40%) completed multiple virtual away rotations. Among the 35 applicants who completed a virtual away rotation, the mean number of virtual away rotations was 1.61 (SD 1.1). There were no differences in Alpha Omega Alpha status (*P* = .77), Gold Humanism Honor Society status (*P* = .52), honors in otolaryngology clerkship (*P* = .11), mean number of honored clerkships (*P* = .26), class quartile (*P* = .08), USMLE step 1 score (*P* = .46), USMLE step 2 score (*P* = .83), number of abstracts, posters, or presentations (*P* = .28), volunteer experiences (*P* = .22), leadership positions (*P* = .24), percentage couples matching (*P* = .49), or percentage taking a research year (*P* = .39) between those who did and did not complete a virtual away rotation (Table 1).

**Table 1:** Applicant Characteristics

Variable	Virtual away, N = 35	No virtual away, N = 80	*P* value
AΩA—number (%)			.77
No	18 (51.4)	38 (47.5)	
Yes	13 (37.1)	35 (43.8)	
No chapter	4 (11.4)	7 (8.8)	
GHHS—number (%)			.52
No	25 (71.4)	62 (77.5)	
Yes	5 (14.3)	12 (15.0)	
No chapter	5 (14.3)	6 (7.5)	
Honors in the specialty applied to—number (%)			.11
No	2 (6.7)	0 (0.0)	
Yes	28 (93.3)	59 (100.0)	
Number of clerkship honors—mean (SD)	4.5 (2.4)	4.0 (2.4)	.26
Cumulative quartile—number (%)			.08
First	14 (58.3)	46 (74.2)	
Second	8 (33.3)	7 (11.3)	
Third	1 (4.2)	7 (11.3)	
Fourth	1 (4.2)	2 (3.2)	
Step 1—mean (SD)	249.5 (11.5)	247.3 (11.4)	.46
Step 2—mean (SD)	256.6 (9.9)	256.0 (10.0)	.83
Abstracts, posters, and presentations—mean (SD)	8.6 (3.1)	7.7 (3.6)	.28
Publications—mean (SD)	6.1 (3.3)	4.8 (3.3)	.06
Research experiences—mean (SD)	6.7 (2.3)	6.5 (2.8)	.45
Volunteer experiences—mean (SD)	8.0 (2.7)	7.3 (2.7)	.22
Leadership positions—mean (SD)	5.7 (3.2)	5.0 (2.8)	.24
Couples match—number (%)			.49
No	31 (88.6)	74 (92.5)	
Yes	4 (11.3)	6 (7.5)	
Research year—number (%)			.39
No	27 (77.1)	67 (83.8)	
Yes	8 (22.9)	13 (16.3)	

Abbreviations: AΩA, Alpha Omega Alpha; GHHS, Gold Humanism Honor Society.

### Interview and Match Outcomes

The mean interview rate (total interview offers/total applications submitted) was 27.8% (SD 29.7) for the entire sample (Table 2). The overall match rate was 72.2%. Among applicants who completed at least 1 virtual away rotation, 60% of the virtual away rotations were followed by a subsequent interview offer (virtual away rotation yield). Among applicants who completed at least 1 virtual away rotation, 7.6% of their total interview offers came from programs where they had completed a virtual away rotation (virtual away rotation to interview ratio) and 4 (11.4%) matched at a program where they completed a virtual away rotation.

**Table 2:** Overall Interview and Match Outcomes

Variable	N = 115
Virtual away rotation yield^a^—mean (SD)	60.0 (44.5)
Virtual away rotation interview ratio^a^—mean (SD)	7.6 (7.0)
Interview rate—mean (SD)	27.8 (29.7)
Matched—number (%)	83 (72.2)
Matched at virtual away rotation^a^—number (%)	4 (11.4)

^a^ Among 35 applicants who completed at least 1 virtual away rotation.

Applicants who completed a virtual away rotation had a significantly higher overall number of interview offers (mean [SD]: 14.9 [8.2] vs 11.6 [7.9]; *P* = .03) but also applied to a higher mean number of programs (81.5 [26.5] vs 70.4 [36.2], *P* = .21). There was no significant difference in the overall rate of interview offers relative to programs applied (mean [SD]: 23.1% [21.6] vs 29.9% [32.5]; *P* = .97) or match success (82.9% vs 67.5%; *P* = .09) between those who did and did not complete a virtual away rotation at a specific program (Table 3).

**Table 3:** Comparison of Applicant Outcomes by Whether They Completed a Virtual Away Rotation

Variable	Virtual away, N = 35	No virtual away, N = 80	*P* value
Match, number (%)			.09
Yes	29 (82.9)	54 (67.5)	
No	6 (17.1)	26 (32.5)	
Interview rate, mean (SD)	23.1 (21.6)	29.9 (32.5)	.97
Total interviews, mean (SD)	14.9 (8.2)	11.6 (7.9)	.03
Total programs applied, mean (SD)	81.4 (26.5)	70.4 (36.2)	.21

**Figure 1:**
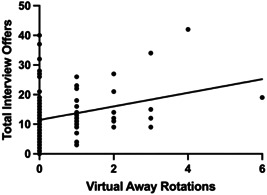
Univariable ordinary least square regression model for the number of virtual away rotations predicting the total number of interviews.

In an OLS regression model, the number of virtual away rotations was positively correlated with the number of interview offers (Figure 1). Specifically, on average each additional virtual away rotation was associated with an increase in at least 2 additional interview offers (*β* coefficient: 2.29 [95% confidence interval, CI: 0.8‐3.7; *P* < .01]). In a logistic regression model assessing the odds of match success was not significantly different for applicants doing at least 1 virtual away rotation compared to none (odds ratio, OR: 2.3, 95% CI: 0.9‐6.3; *P* = .09) (Table 4).

**Table 4:** Logistic Regression Model for Odds of Match Success

	OR	95% CI	*P* value
Matching			
0 virtual aways	Reference	Reference	Reference
1+ virtual away	2.3	(0.9, 6.3)	.09

Abbreviations: CI, confidence interval; OR, odds ratio.

### Application‐Level Analysis

The 115 otolaryngology applicants in this sample submitted a total of 8478 applications, among which 59 applications were to programs where a virtual away rotation was completed. Applicants to programs where a virtual away rotation was completed were significantly more likely to receive an interview offer (62.7% vs 16.8%, *P* < .01) and to match at that program (6.8% vs 0.9%; *P* < .01) compared to applicants who had not performed a virtual away at the program (Table 5). When limiting the analysis to only applications that resulted in an interview offer (n = 1450), there was no significant difference in the percentage of applications that resulted in a match between those who did and did not complete a virtual away rotation at that program (10.8% vs 5.6%; *P* = .17).

**Table 5:** Application‐Level Analysis for Interview and Match Outcomes

Outcome	The applicant completed virtual away at the program, N = 59	The applicant did not complete virtual away at the program, N = 8419	*P* value
Among 8478 applications to all programs			
Interviewed at program—number (%)	37 (62.7)	1413 (16.8)	<.01
Matched at program—number (%)	4 (6.8)	79 (0.9)	<.01

Outcome	The application includes virtual away at program, N = 37	The application does not include virtual away at the program, N = 1413	*P* value
Among 1450 applications to programs that resulted in interviews			
Matched at program	4 (10.8)	79 (5.6)	.17

Applicants who completed a virtual away rotation had significantly increased odds of receiving an interview offer (OR: 8.4, 95% CI: 4.9‐14.2; *P* < .01) and matching (OR: 7.7, 95% CI: 2.7‐21.7; *P* < .01) at the program where they completed the virtual away rotation, based on logistic regression models (Table 6). However, performing a virtual away rotation was not associated with an increased likelihood of matching when the analysis was limited to applicants who received interview offers (*P* = .18).

**Table 6:** Odds of Receiving an Interview Offer or Matching at a Program Where a Virtual Away Rotation was Completed

Outcome	Odds ratio	95% Confidence interval	*P* value
Among all applications (n = 8478)			
Interview offer	8.4	4.9, 14.2	<.01
Matching	7.7	2.7, 21.7	<.01
Among applications that received an interview offer (n = 1450)			
Matching	2.1	0.7, 5.9	.18

## Discussion

In this study, we found that nearly one‐third of otolaryngology applicants completed at least 1 virtual away rotation and that completing a virtual away rotation was associated with significantly increased odds of receiving an interview and matching at that specific program of interest compared to applicants not completing a virtual away rotation. When limiting the analysis to only applicants who received interview offers at a specific program, having done a virtual away rotation was not associated with an incremental increase in odds of match success. As away rotations evolve and the virtual landscape continues to expand, there are potential roles for virtual, in‐person, and hybrid away rotation options, analogous to how in‐person and telehealth have melded in clinical practice. The present study affords relevant insights and might help inform otolaryngology residency applications and recruitment strategies.

The virtual away rotations that debuted during the COVID‐19 pandemic fostered connections with applicants and mirrored some of the didactic and experiential aspects of in‐person away rotations but varied widely in length, structure, and content. Programs in several surgical specialties including otolaryngology,^10^, ^11^, ^12^ orthopedic surgery,^13^, ^14^ plastic surgery,^15^ urology,^16^, ^17^, ^18^ and vascular surgery^19^ have described their virtual away rotations and reported survey results from participating applicants. Some common activities included in the virtual away rotations were social hours with the residents, faculty meet‐and‐greets, grand rounds presentations, journal clubs, research presentations, didactic curricula, and question‐and‐answer sessions. One plastic surgery program even described a “virtual OR” component where cases were live streamed for participants and involved both pre‐ and post‐op case discussions.^15^

Student evaluations of virtual away rotations have overall been favorable, with resident/faculty interactions and learning about the program often considered to be 2 of the most valuable aspects.^10^, ^11^, ^14^, ^19^ Faculty and residents have responded favorably as well, finding virtual rotations useful for assessing student characteristics such as knowledge base and communication skills.^14^ Common challenges reported by faculty included a limited ability to assess an applicant's fit for the program, lack of hands‐on experiences, and an increase in personal workload.^16^, ^18^, ^19^, ^20^

In addition to facilitating connections between students and programs, virtual away rotations may also serve as an informal “signal” of an applicant's interest in a training program. In a recent analysis of otolaryngology preference signaling outcomes in the 2020 to 2021 and 2021 to 2022 application cycles, we found that on average 59.5% of preference signals resulted in an interview at that program.^21^ Similarly, in this study, we found that on average 60% of virtual away rotations resulted in an interview offer. In a market where on average otolaryngology programs receive 300 to 500 applications per year,^22^ virtual away rotations may be another way for applicants to distinguish themselves.

Although virtual away rotations cannot reproduce all aspects of the in‐person experience, they offer a more affordable and more equitable alternative. In a recent cost analysis of the otolaryngology residency application process, we found an average decrease in spending of $5669 per applicant in the 2020 to 2021 application cycle, presumably due to a lack of in‐person away rotations and interviews that year.^23^ Both applicants and programs have emphasized the ability for virtual away rotations to help level the playing field for applicants from economically disadvantaged backgrounds, those without a home program, those who identify as URiM, and those who are IMG.^3^, ^24^, ^25^ Our findings suggest that virtual away rotations might help applicants with interviewing and matching at a program of interest if virtual rotations are offered. Virtual away rotations may have a role that long outlasts the COVID‐19 pandemic, but their success will require the investment of resources and identification of best practices for enrollment access, structured didactic curricula, and assessment.

Although most residency programs that offered virtual away rotations early in the pandemic have returned to an in‐person away rotation model, we anticipate a growing role for virtual interactions in the residency application process, whether as formal away rotations or more focused experiences.^26^ In clinical care, the meteoric rise in telehealth at the start of the pandemic was followed by a precipitous drop in use once clinics reopened, but there is growing evidence of long‐term adoption.^27^ Whether in‐person away rotations and virtual away rotations can co‐exist remains an open question. One potential solution to promote equity would be to permit applicants to complete 1 in‐person away rotation per cycle and any number of virtual aways. Ideally, this approach would incorporate funding opportunities to help cover the costs of in‐person away rotations for applicants with financial needs.

This study has several limitations. There is significant heterogeneity among virtual rotations in terms of their structure, length, and content. For example, some virtual rotations may have better allowed applicants to demonstrate their skills to faculty while others may have been more educational focused, and we were unable to capture these nuances in our analysis. The sample size of 115 otolaryngology applicants is only 18.1% of the total 2021 otolaryngology applicant pool reported by ERAS,^22^ so the external validity is limited and these results may not be generalizable to all otolaryngology applicants. Furthermore, fewer than a third of these applicants participated in virtual away rotations. As a result, some analyses may have been underpowered. In addition, it is unknown how many programs or which programs offered virtual away rotations. Larger programs with more resources may have been more likely to offer away rotations, and individuals who opted to engage with these offerings may have differed from their peers who opted not to do so, creating the potential for selection bias. The Texas STAR database was not distributed to international medical schools, so we were unable to assess outcomes for IMG's, who are key stakeholders in the conversation about virtual away rotations. Additionally, there may be recall bias as applicants completed the survey in April 2021 which was approximately 7 months after submitting their applications. Despite these limitations, this exploratory study provides novel data on the effects of virtual away rotations that can be used to guide further investigation and decision making.

## Conclusions

Virtual away rotations were completed by approximately one‐third of otolaryngology applicants in the 2021 match year and were associated with significantly increased chances of receiving an interview and matching at the programs of interest.

## Author Contributions

**Nicholas R. Lenze**, study design, data interpretation, manuscript drafting; **William J. Benjamin**, study design, analysis, data interpretation, manuscript drafting; **Lauren A. Bohm**, study design, data interpretation, manuscript revision, project supervision; **Marc C. Thorne**, study design, data interpretation, manuscript revision, project supervision; **Michael J. Brenner**, study design, data interpretation, manuscript revision, project supervision; **Angela P. Mihalic**, data acquisition, manuscript revision, project supervision; **Robbi A. Kupfer**, study design, data interpretation, manuscript revision, project supervision.

## Disclosures

### Competing interests

The authors declare that they have no competing interests.

### Funding source

None.
